# A community-based comprehensive intervention to reduce syphilis infection among low-fee female sex workers in China: a matched-pair, community-based randomized study

**DOI:** 10.1186/s40249-019-0611-z

**Published:** 2019-12-03

**Authors:** Wei Dong, Chu Zhou, Ke-Ming Rou, Zun-You Wu, Jun Chen, Sarah Robbins Scott, Man-Hong Jia, Yue-Jiao Zhou, Xi Chen

**Affiliations:** 1 Division of HIV Prevention, National Center for AIDS/STD Control and Prevention, Chinese Center for Disease Control and Prevention, 155 Changbai Road, Beijing, 102206 China; 2 Department of Epidemiology, UCLA Fielding School of Public Health, Los Angeles, USA; 3 Institute of AIDS/STD Control and Prevention, Yunnan Provincial Center for Disease Control and Prevention, Kunming, China; 4 Institute of AIDS Control and Prevention, Guangxi Zhuang Autonomous Region for Disease Control and Prevention, Nanning, China; 5 Division of AIDS/STD Control and Prevention, Hunan Provincial Center for Disease Control and Prevention, Changsha, China

**Keywords:** China, Female sex workers, Human immunodeficiency virus, Syphilis

## Abstract

**Background:**

Low-fee female sex workers (FSWs) are at high risk of acquiring and spreading human immunodeficiency virus (HIV)/sexually transmitted diseases (STDs) in China. There is an urgent need to develop comprehensive intervention measures targeted towards low-fee FSWs to reduce HIV/STD infections. Thus, this study aimed to reduce HIV/STD infections among low-fee FSW through a matched-pair, community-based randomized intervention trial carried out in 12 cities in three provinces in China.

**Methods:**

Four cities from Guangxi Zhuang Autonomous Region, four from Yunnan Province, and four from Hunan Province were paired and participants received either the intervention package (including condom promotion, HIV and syphilis testing, reimbursement for syphilis treatment costs, and free anti-retroviral therapy or the current standard of care. Venue-based, convenience sampling was used to recruit FSWs. A face-to-face interview and HIV and syphilis blood testing was conducted at baseline and follow-up intervals of 24 months. Generalized linear mixed models (GLMM) were used to evaluate the effect of the intervention package on reducing HIV/STD infection in the FSWs.

**Results:**

A total of 1024 eligible FSWs were enrolled in the baseline survey and 843 in the follow-up. GLMM results showed that syphilis infection was reduced by 49% in the intervention group compared to the current standard of care group (*P* = 0.0378, *OR* = 0.51, 95% *CI*: 0.27–0.96). FSWs aged 35 years or older were 2.38 times more likely to get syphilis infection compared to those younger than 35 years old (*P* <  0.0001, *OR* = 2.38, 95% *CI*: 1.55–3.65). The risk of syphilis infection among more educated FSWs was 0.43 times less than those with lower levels of education (*P* <  0.05, *OR* = 0.43, 95% *CI*: 0.63–0.93).

**Conclusions:**

This study demonstrates that comprehensive interventions can lead to significant declines in syphilis infection amongst low-tier FSWs. Integrating both behavioral and biomedical intervention measures should be considered when developing programs for low-fee FSWs.

**Trial registration:**

CHiCTR-TRC-12002655.

## Multilingual abstracts

Please see Additional file 1 for translations of the abstract into the five official working languages of the United Nations

## Background

Female sex workers (FSWs) are at high risk of acquiring human immunodeficiency virus (HIV) and sexually transmitted infections (STIs), and transmitting HIV and STIs to the general population [1]. FSWs are 13.5 times more likely to be infected with HIV compared to the general female population in low- and middle-income countries [2]. Although commercial sex work remains illegal in China, it has grown significantly since re-emerging in the early-1980s [3]. In 1995, the Chinese government recognized FSWs as a key population in the fight against HIV/acquired immunodefiency syndrome (AIDS) and began implementing prevention programs aimed at improving condom use and promoting voluntary counselling and testing amongst this group [3]. FSWs were also included in national sentinel surveillance [4], when, in 2015, the number of sentinel cities was increased to 520 sites across 31 provinces (China’s 2011–2015 national HIV/AIDS sentinel surveillance report unpublished data).

Sex work in China is organized and influenced by different socio-economic factors, ranging from low-fee to high-fee FSWs [5]. Several tiers of FSWs have been delineated in the literature according to price charged, work venues, and prestige [5–7]. In China, the distinction between low-fee and high-fee FSWs is based on two factors. First, the price, that is, the charge of 80 Yuan (RMB, the currency in China) or less per commercial sex act is defined as low-fee FSWs, while the high-fee and medium-fee FSWs usually charge more than RMB 80 per commercial sex act. Second, the venue factor, the type of venue where high-fee and medium-fee FSWs work, mainly refers to large clubs, karaoke television (KTV), high-grade bath centers. Low-fee FSWs typically work out of poorer areas and establishments such as small salons/massage rooms, small guesthouses/inns, self-rented rooms, temporary rental housing during market days, or on the street [8]. Compared to mid- or high-fee FSWs, low-fee FSWs are typically of a lower socio-economic status, have less education, poorer hygiene practices, and are highly mobile due to being more frequently targeted by police [3]. Low-tier FSWs are more likely to have a greater number of clients, a lack of understanding of HIV and other STIs, and exhibit the lowest rates of condom use with both commercial and non-commercial partners [7–9]. They also often lack an “organizer”, who can help ensure their safety and support their engagement in intervention measures [10].

Data from previous studies show that low-fee FSWs have significantly higher HIV and syphilis prevalence compared to higher-fee FSWs. A meta-analysis conducted in six Chinese cities in 2009 found that the HIV prevalence among low-fee FSWs was 1.37%, yet only 0.28 and 0.07% among middle- and high-fee FSWs, respectively [9]. A cross-sectional study conducted in 12 Chinese sites in 2012 targeting low-fee FSWs showed that HIV prevalence for this sample was much higher, at 4.7% [8]. According to China’s 2011–2015 national HIV/AIDS sentinel surveillance report, compared with middle- and high-fee FSWs, low-fee FSWs exhibit higher HIV and syphilis prevalence (HIV: 0.1–0.2% in middle- and high-fee FSWs vs 0.4–0.6% in low-fee FSWs; Syphilis: 1.6–1.9% in middle- and high-fee FSWs vs 3.6–4.6% in low-fee FSWs, unpublished data, respectively) [11].

A substantial amount of research in the past 20 years has demonstrated the critical importance of condom promotion, testing and counseling, treatment and prevention, and social support needs of this high-risk group as well as best practices for implementing FSW-targeted interventions in China [3, 12, 13]. But, multi-site, community-based studies for this population have been very limited. In a review of 129 HIV/STI prevention intervention studies targeting FSWs in China between 2000 and 2013, 126 (98.4%) studies employed a pre/post design with an open or closed cohort. But only three studies were carried out in multiple provinces [3]. Also, few studies focused on exploring comprehensive interventions to reduce HIV/STI infection among low-fee FSWs [13], since low-fee FSWs are harder to reach. Hence, our study evaluated the effectiveness of a comprehensive intervention package in reducing syphilis infection among FSWs in 12 cities in China using a matched-pair, community-based randomized trial design.

## Methods

### Study design and city selection

This study is a matched-paired, community-based randomized trial. Twelve cities were selected from two southern provinces (Guangxi Zhuang Autonomous Region and Yunnan) and a central province (Hunan) in China. Four cities in the same province were allocated into two intervention cities and two current standard of care cities according to parallel control design, and the ratio of intervention city and current standard of care city was 1:1.

The three provinces were selected based on the following selection criteria: (1) among the top five provinces with the highest proportion of HIV cases acquired through heterosexual transmission reported to the national surveillance system in 2012 (91.8% in Guangxi, 81.8% in Hunan, and 79.8% in Yunnan); (2) HIV prevalence of greater than 2% among low-fee FSWs according to data from the HIV sentinel surveillance survey in 2012, and (3) local outreach workers had experience conducting HIV/STI interventions for low-fee FSWs.

### Randomisation and matched-pair criterion

Randomization was embodied in the allocation of intervention cities and current standard of care cities using stratified block randomization. To minimize the underlying regional disparity and possible inter-group contamination, we defined the matching rules of the paired cities as follows: (1) each pair was located in the same province and geographically separated by at least 100 km and (2) local FSWs’ syphilis infection baseline rates were not significantly different between the paired cities. After stratification according to baseline syphilis infection, we used a coin toss to determine the random allocation scheme.

A total of six paired cities were included in our study. The intervention cities included: Zhangjiajie and Jianghua in Hunan Province; Liuzhou and Pingnan in Guangxi Autonomous Region; and Dali and Jinghong in Yunnan Province. The current standard of care cities included: Jishou and Lingling in Hunan Province; Guigang and Du’an in Guangxi Autonomous Region; and Kaiyuan and Menghai in Yunnan Province.

Since the research design was a community-based, cluster randomized trial, once the nature of the cluster was determined, the activities were implemented to the entire city, thus research participants were not randomized. Moreover, the research design team and the field implementation team were two independent teams, without any intersection in the grouping and allocation process. The randomization was carried out entirely by the study design team, while the field implementation teams were not aware of their city’s allocation until the intervention began. To sum, the randomization scheme was generated by the research team and the field implementation team was responsible for the participant recruitment.

### Sampling method and inclusion criteria

Venue-based convenience sampling was used to recruit FSWs. During the pre-research stage from 1st to 30th September 2012, local outreach workers (including Center for Disease Control [CDC] workers, workers from community-based hospitals, and community-based organizations [CBO]) went to every low-fee venue they could access, including small beauty salons/massage parlors, small inns/guesthouses, self-rented houses and temporary rental housing during market days, to investigate the number of low-fee FSWs in those venues. According to this number, it was estimated that a sample size of 100 eligible FSWs per city was feasible to enroll in the study. Inclusion criteria for the study was as follows: (1) 18 to 60 years of age, (2) self-identifying as a FSW, and (3) charging less than RMB 80 (approximately USD 12 [United States Dollars]) per vaginal sex act [8]. The exclusion criteria were previous HIV-positive individuals before the survey and drug users.

### Study procedure

#### Baseline survey

Prior to initiation of the study, we made adequate preparations for the personnel, study materials, and investigation methods. After the clinical trial registration was approved, we began to formally recruit study subjects on November 9th, 2012. The baseline survey was conducted from 9th November 2012 to 31st January 2013. Trained local outreach workers who were familiar with the local dialect introduced the study procedure to eligible low-fee FSWs. Low-fee FSWs who agreed to participate in the study signed the informed consent themselves. Local outreach workers conducted face-to-face interviews with all participants for about 30 min in a private room. Interview topics included socio demographic characteristics, HIV/STI related knowledge and awareness, condom use, and other information regarding sex trade. Then after pre-test counseling, five milliliters (ml) of venous blood were collected from each participant to be tested for HIV and syphilis.

HIV screening was conducted with enzyme-linked immunosorbent assays (ELISA) (Kinghawk Pharmaceutical Co., Ltd., Beijing, China), syphilis screening was performed using Rapid Plasma Reagin (RPR) tests (Kinghawk Pharmaceutical Co., Ltd., Beijing, China) and then confirmed by a Treponema Pallidum Particle Agglutination (TPPA) assays (Serodia-TPPA, Fujirebio Inc., Tokyo, Japan). The participants received their test results via telephone calls by CDC outreach workers. Those who were HIV or syphilis positive received further testing and counseling at the local CDC and were referred to the national HIV treatment program, or to STI treatment clinics which cooperated with the local CDC.

#### Community-based intervention activities

The intervention was conducted between May 2013 and May 2015. In intervention cities, a comprehensive intervention package was implemented in all FSW- working venues, which local outreach workers could access. The intervention package included condom promotion, HIV and syphilis testing, reimbursement for syphilis infection treatment, and antiretroviral therapy (ART). A complete description of the intervention package can be found in the supplementary material (see Additional file 2). The overall intervention period was 24 months.

#### Current standard of care group

In current standard of care cities, FSWs recieved routine outreach activities, which included condom distribution, annual HIV/syphilis testing, and referral for HIV/STI infection.

#### Follow-up survey

A follow up survey was carried out from 1st May 2015 to 31st July 2015. The procedure was similar to the baseline survey. The identical venues covered by the baseline survey were investigated and all FSWs in the venues were recruited in the follow-up survey. A face-to-face interview and HIV and syphilis blood collection and testing were conducted. Due to high mobility, not all FSWs in the baseline survey were captured in the follow-up survey. Also, we were unable to match the follow-up and baseline subjects to the same person.

### Statistical analysis

The primary outcomes were syphilis infection rate, newly-diagnosed HIV infection rate, and consistent condom use rate. Secondary outcomes included HIV-related knowledge awareness rate and self-reported rate of STD symptoms in past six months. These outcome measures were based on blood test results and questionnaire analysis obtained during baseline and follow-up surveys.

Data were double-entered in EpiData software (version 3.02, the EpiData Association, Odense, Denmark) and analysed using SAS software for Windows (version 21. SAS Institute Inc., Cary, NC, USA,). At the individual level, for the baseline and follow-up surveys, the characteristics of FSWs were described. For categorical variables, percentages and frequencies were presented. Chi-square analysis was applied to examine differences in characteristics between the baseline and follow up surveys. At the city level, paired *t*-test was used to compare the rate of syphilis infection between the cities where the intervention package was implemented and the cities receiving the current standard of care in the baseline and follow-up survey.

We used individual-level analysis accounting for clustering to evaluate our main outcome of syphilis infection [14]. The baseline and follow-up datasets were combined and generalized linear mixed models (GLMM) were applied to detect the intervention effect accounting for both individual-level and cluster-level [15, 16]. Because of the time changing trends of some key variables, a time variable was introduced into the model. The interaction of the time and grouping was determined to be the major analysis factor which represented the net impact of the intervention by controlling the time effect [17]. The statistical principle was based on the formula as follows: $\frac{{\left({\mathrm{OR}}_{\mathrm{time}=1/\mathrm{time}=0}\right)}_{group=1}}{{\left({\mathrm{OR}}_{\mathrm{time}=1/\mathrm{time}=0}\right)}_{group=0}}={e}^{\beta_3}.{\beta}_3$ is the interaction term coefficient, which can be interpreted as the natural logarithm of the ratio between the risk of an event (*OR*) before and after the intervention package in the intervention group and the risk of an event (*OR*) before and after the intervention in the current standard of care group [18].

Based on the results of a previous literature review and the results of a single-covariate binary logistic regression, we included six variables into the GLMM model. The interaction of time and grouping, FSWs’ age, educational level, price charged per vaginal sex act, and daily number of clients were defined as fixed effects and city as a random effect. Odds ratios (*OR*) with 95% confidence intervals (*CI*) were estimated, and a *P* value ≤0.05 was considered statistically significant. Newly-diagnosed HIV infection, self-reported STI symptoms, condom use and HIV–related knowledge were also analyzed in the GLMM because they are major outcome variables and process variables [19].

### Ethical approval

The study was approved by the Institutional Review Board of the National Centre for AIDS/STD Control and Prevention of the Chinese Center for Disease Control and Prevention (The approval number is X120717225). A signed, informed consent form was obtained from all participants. Each participant was paid 50 RMB for participation in the baseline and follow-up surveys.

## Results

**Fig. 1:**
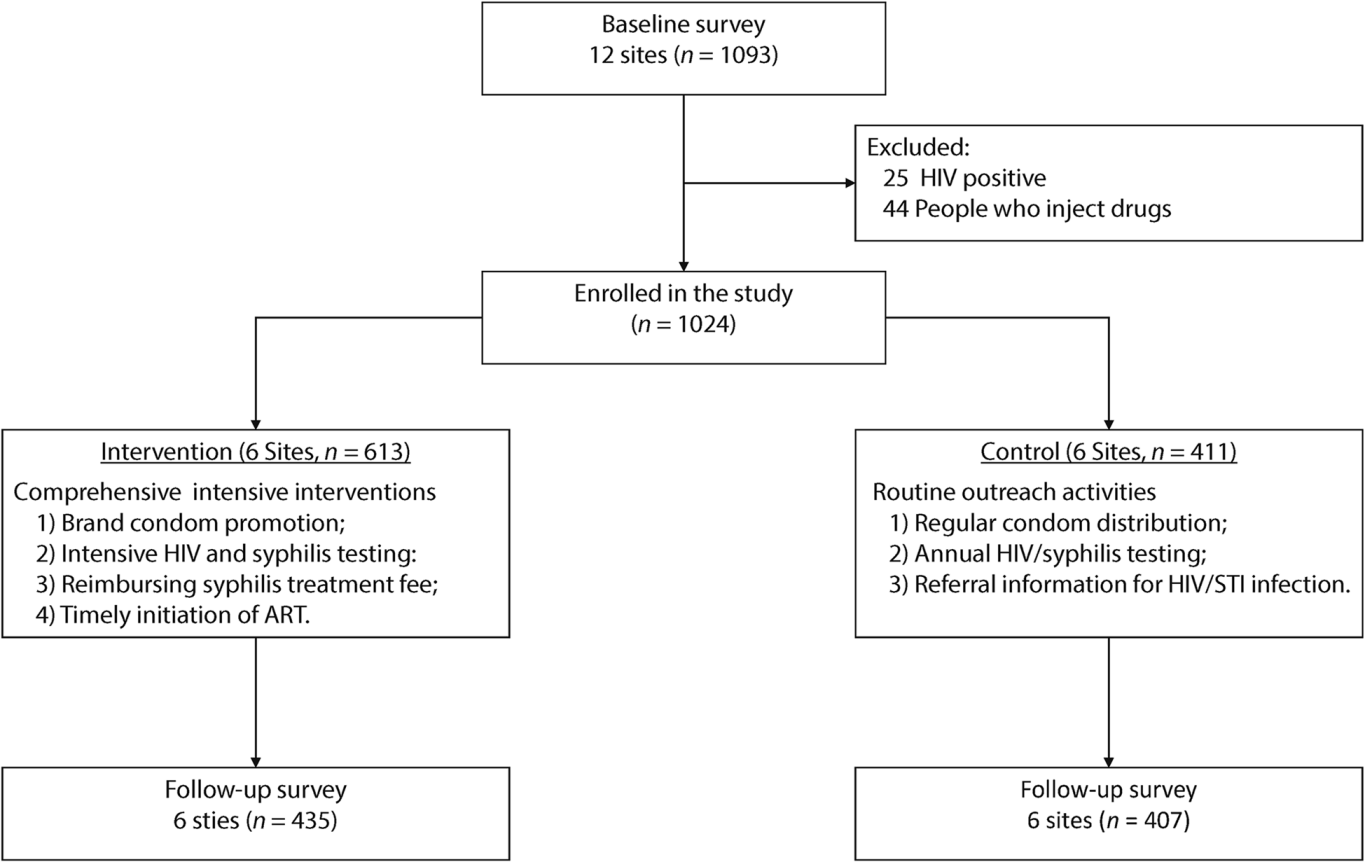
Flowchart of study procedure. HIV: Human immunodeficiency virus; STI: Sexually transmitted infection

A total of 1093 FSWs completed the questionnaire and blood collection during the baseline survey between November 2012 and January 2013. Among them, 25 FSWs confirmed previous HIV positive and 44 reported drug use history and were excluded. The sample size at baseline for those receiving the intervention package was 613 and those receiving the current standard of care (i.e., control group) was 411. In the follow-up survery, 435 participants receiving the intervention package and 407 FSWs received the current standard of care **(**Fig. 1**)**. In both surveys, FSWs were more likely to be over 35, mostly married or cohabiting, and most were educated less than five years.

### Individual level differences between intervention and control groups in baseline and follow-up

Comparisons of FSWs’ characteristics between those receiving the intervention package and those receiving the current standard of care during baseline and follow-up surveys are shown in Table 1. For the major outcome variables, such as condom use (*P* = 0.917), self-reported STD symptoms in past six months (*P* = 0.616), newly-diagnosed HIV infections (*P* = 0.340), and syphilis infection (*P* = 0.076), the intervention and current standard of care populations were comparable. In the follow-up survey, the proportion of FSWs older than 35 and single/divorced/widowed was slightly higher than the baseline population. Compared to the current standard of care group, the rate of consistent condom use in those receiving the intervention package was higher (77.0% vs 70.5%, *P* = 0.032). The syphilis infection rate in cities where the intervention package was implemented was significantly lower than in the cities which received the current standard of care (6.2% vs 15.2%, *P* <  0.001).

**Table 1:** Demographic characteristics of FSWs in intervention and standard of care groups at baseline and follow-up

Variables	Baseline			Follow-up		
	Intervention *N* (%)	Current standard of care *N* (%)	*P* value	Intervention *N* (%)	Current standard of care *N* (%)	*P* value
**Age group**						
Age < 35	219 (35.7)	204 (49.6)	< 0.001	110 (25.3)	186 (45.7)	< 0.001
Age ≥ 35	394 (64.3)	207 (50.4)		325 (74.7)	221 (54.3)	
**Marital status**						
In marriage/Cohabitation	441 (71.9)	294 (71. 5)	0.887	278 (63.9)	272 (66.8)	0.373
Single/Divorced/widowed	172 (28.1)	117 (28.5)		157 (36.1)	135 (33.2)	
**Educational level** ^**a**^						
Less than or equal to primary school	389 (63.6)	235 (57.2)	0.040	268 (61.6)	230 (57.2)	0.196
Higher than primary school	223 (36.4)	176 (42.8)		167 (38.4)	172 (42.8)	
**Daily number of clients** ^**a**^						
Less than five	448 (73.2)	336 (81.8)	0.002	362 (83.2)	311 (78.1)	0.064
More than five	164 (26.8)	75 (18.2)		73 (16.8)	87 (21.9)	
**Venue type** ^**a**^						
Self-rented rooms and “market day” rooms	203 (33.1)	144 (35.0)	0.524	208 (47.8)	196 (49.4)	0.654
Small salon/massage room/ guesthouse/inn	410 (66.9)	267 (65.0)		227 (52.2)	201 (50.6)	
**Price charged per vaginal sex**						
≤ RMB 50 **	487 (79.4)	253 (61.6)	< 0.001	261 (60.0)	179 (44.0)	< 0.001
> RMB 51	126 (20.6)	158 (38.4)		174 (40.0)	228 (56.0)	
**HIV-related knowledge awareness**						
Knowledgeable	427 (69.7)	251 (61.1)	< 0.001	409 (94.0)	377 (92.6)	0.418
Non-knowledgeable	186 (30.3)	160 (38.9)		26 (6.0)	30 (7.4)	
**Consistent condom use**						
Yes	344 (56.1)	232 (56.4)	0.917	335 (77.0)	287 (70.5)	0.032
No	269 (43.9)	179 (43.6)		100 (23.0)	120 (29.5)	
**Self-reported STD symptoms in past 6 months**						
Yes	64 (10.4)	47 (11.4)	0.616	9 (2.1)	30 (7.4)	< 0.001
No	549 (89.6)	364 (88.6)		426 (97.9)	377 (92.6)	
**Newly-diagnosed HIV infection**						
Positive	11 (1.8)	11 (2.7)	0.340	3 (0.7)	4 (1.0)	0.641
Negative	602 (98.2)	400 (97.3)		432 (99.3)	403 (99.0)	
**Syphilis infection**						
Positive	68 (11.1)	61 (14.8)	0.076	27 (6.2)	62 (15.2)	< 0.001
Negative	545 (88.9)	350 (85.2)		408 (93.8)	345 (84.8)	

**RMB: Short for renminbi, is the currency issued by the Chinese government; HIV: Human immunodefiency virus; STD: Sexually transmitted diseases; FSW: Female sex workers

*P <  0.01

a: Some objects had no response to this variable

### Cluster level differences of syphilis infection

**Fig. 2:**
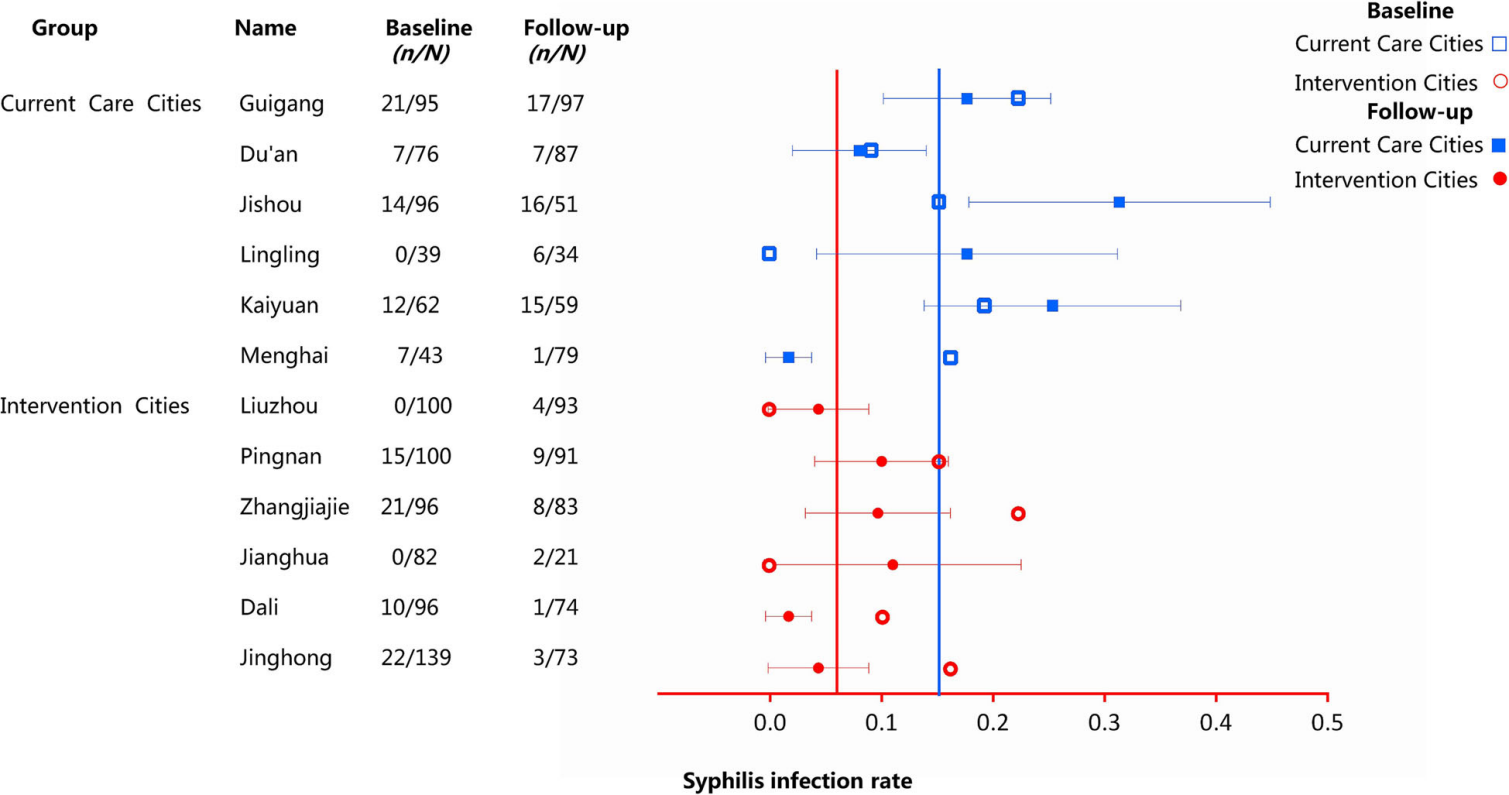
Syphilis rates in intervention and current standard of care cities at baseline and follow-up. Comparison of syphilis infection rates by city. The hollow scatters represent the syphilis infection rates during the Baseline survey, while the solid scatter and corresponding interval line represent the syphilis infection rate with 95% CI of the follow up survey. Vertical reference lines show the mean syphilis infection rate in the cities where the intervention was implemented (red) and the cities receiving the current standard of care (blue) in follow-up survey. *CI*: Confidence intervals

In paired *t*-test statistical analysis, there was no significant difference in syphilis infection between intervention cities and current standard of care cities (*P* = 0.264) at the baseline survey, but the difference was significant in the follow-up survey (*P* = 0.016). The overall syphilis infection rate in the current standard of care group was 0.15 times higher than in the cities which received the intervention package **(**Fig. 2**)**.

### GLMM analysis

Results of the GLMM analysis are shown in Table 2**.** Syphilis infection, newly-diagnosed HIV infection, condom use, HIV-related knowledge, and self-reported STD symptoms were taken as outcome variables. The intervention package was negatively correlated with syphilis infection (*P* = 0.0378, *OR* = 0.51, 95% *CI*: 0.27–0.96), which implies that the intervention reduced the risk of syphilis infection by 49% in the cities receiving the intervention package compared to the cities with the current standard of care. Participants in the intervention cities also self-reported fewer STD symptoms (*P* = 0.0135, *OR* = 0.33, 95% *CI*: 0.13–0.79). The results also showed however, that the intervention had no effect on newly-diagnosed HIV infection (*P* = 0.8753, *OR* = 1.15, 95% *CI*: 0.20–6.54), condom use (*P* = 0.2474, *OR* = 1.35, 95% *CI*: 0.81–2.23) and HIV-related knowledge (*P* = 0.5038, *OR* = 0.81, 95% *CI*: 0.43–1.51).

**Table 2:** Odds of syphilis, newly-diagnosed HIV infection, self-reported STD symptoms, condom use and HIV-related knowledge among FSWs in China

	Syphilis infection		Newly-diagnosed HIV infection		Self-reported STD symptoms		Condom use		HIV-related knowledge	
	*OR* (95% *CI*)	*P* value	*OR* (95% *CI*)	*P* value	*OR* (95% *CI*)	*P* value*	*OR* (95% *CI*)	*P* value	*OR* (95% *CI*)	*P* value
**Group**										
Intervention	0.51 (0.27–0.96)	0.0378	1.15 (0.20–6.54)	0.8753	0.33 (0.13–0.79)	0.0135	1.35 (0.81–2.23)	0.2474	0.81 (0.43–1.51)	0.5038
Current standard of care	1.00	–	1.00	–	1.00	–	1.00	–	1.00	–
**Age**										
≥ 35 years	2.38 (1.55–3.65)	< 0.0001	1.97 (0.66–5.94)	0.2268	1.31 (0.85–2.02)	0.2168	0.35 (0.25–0.47)	< 0.0001	1.22 (0.89–1.66)	0.2130
< 35 years	1.00	–	1.00	–	1.00	–	1.00	–	1.00	–
**Educational level**										
Higher than primary school	0.63 (0.43–0.93)	0.0189	0.36 (0.12–1.13)	0.0798	1.05 (0.71–1.57)	0.7983	1.72 (1.31–2.27)	< 0.0001	2.32 (1.72–3.14)	< 0.0001
Primary school or less education	1.00	–	1.00	–	1.00	–	1.00	–	1.00	–
**Price charged per vaginal sex act**										
≤ 50 RMB**	1.37 (0.90–2.08)	0.1419	1.79 (0.55–5.86)	0.3360	0.70 (0.45–1.09)	0.1121	0.29 (0.21–0.40)	< 0.0001	0.59 (0.42–0.82)	0.0019
> 51 RMB	1.00	–	1.00	–	1.00	–	1.00	–	1.00	–
**Daily number of clients**										
More than five	1.31 (0.90–1.91)	0.1619	1.76 (0.78–3.98)	0.1722	1.62 (1.07–2.44)	0.0232	0.45 (0.33–0.61)	< 0.0001	0.88 (0.65–1.20)	0.4288
Less than five	1.00	–	1.00	–	1.00	–	1.00	–	1.00	–

**RMB, short for renminbi, is the currency issued by the Chinese government. HIV: Human immunodefiency virus; STD: Sexually transmitted diseases; *CI*: Confidence intervals; *OR*: Odds ratios; FSW: Female sex workers; ——: the particular variable was the reference variable

* *P* < 0.05 indicates statistical significance

FSWs aged 35 years or older were 2.38 times more likely to get syphilis infection compared with those younger than 35 years old (*P* <  0.0001, *OR* = 2.38, 95% *CI*: 1.55–3.65). More so, the risk of syphilis infection among FSWs with a primary school education or higher was 0.43 times less than those with lower levels of education (*P* <  0.05, *OR* = 0.43, 95% *CI*: 0.63–0.93). In addition, the higher daily number of clients, the more likely FSWs were to report STI symptoms (*P* = 0.0232, *OR* = 1.62, 95% *CI*: 1.07–2.44) **(**Table 2**)**.

## Discussion

This study is the first multi-site, community-based trial to reduce syphilis infection in low-fee FSWs in China. After a comprehensive intervention package was implemented for 24 months, our study demonstrated a significant decline of syphilis infection.

The risk of syphilis infection in FSWs receiving the intervention package was 49% less than those receiving the current standard of care. This implies that our comprehensive intervention package is effective in reducing syphilis infection among low-fee FSWs. On one hand, about 2500 women were tested in intervention cities, approximately 20% more than the current care cities (supplementary material). Given this, more potentially asymptomatic patients may have been found, which would reduce the further transmission of syphilis. On the other hand and perhaps most importantly, timely referral to treatment and higher treatment coverage for syphilis, as provided as a part of the intervention package, may have led to this decline and as such, have important advantages for public health [20]. Providing free or low-cost treatment and intervention services to patients infected with syphilis at clinics has been recommended as core components in syphilis control programs in China [21]. This study followed these guidelines in the development of the intervention package, which was found to be effective in reducing the prevalence of syphilis. Thus, current interventions are effective in reducing the source of syphilis infection and reducing the pool of infected individuals.

FSWs need unique attention in order to get access to necessary health services [22]. Previous research has cited that the most common barriers to service utilization by female sex workers are not knowing where the testing sites are located, inconvenient location of clinics, lack of confidentiality, discrimination by health care providers, stigma, and financial burden [22–24]. As such, the intervention provided in this study was tailored to address these concerns. The intervention included one-to-one accompanied referrals and reimbursement of treatment expenses. In cities where the intervention was implemented, 73.3% of syphilis-infected FSWs were successfully referred to the designated STI clinics for further diagnosis and treatment (supplementary material), indicating higher efficiency of outreach services delivered by effective referral activities [21]. More so, self-reported rates of STD symptoms also declined in the intervention cities. Previous research has shown that promotion of consistent condom use coupled with HIV and STD during outreach interventions have increased FSW’s risk awareness to some extent [22, 23, 25]. Additionally, FSWs receiving referral therapy may have contributed to this decline.

Within this study, the comprehensive intervention measures of condom distribution, testing promotion, and timely referral to HIV treatment were utilized in order to impact HIV infection control. As seen in the supplementary material, the intervention group accessed services at a higher rate compared to the current standard of care group. The number of individuals tested for HIV, those using condoms consistenly, and the rate of ART initiation was higher than in the control group. Thus, a comprehensive package promoting both testing and timely treatment referral can be useful HIV prevention among low-tier FSWs. The intervention package, however, did not have a significant effect on condom use, the number of new HIV infections, or HIV-related knowledge. As the HIV epidemic in China is transitioning to one of sexual transmission, authorities have intensified prevention programs to control sexual transmission nationwide. The progress of routine AIDS prevention work in current care cities during the research period may have narrowed the gap with cities where the intervention package was implemented. The decline in the syphilis infection rate in those receiving the intervention may be due to reimbursement of treatment and reduced cost burden of treatment, rather than HIV–related knowledge awareness and condom use promotion. Knowledge and behavior change are slower processes, taking some time before any impact on infection rates may be seen [25]. Additional follow-up at a later time assessing these high risk behaviors and impact on HIV prevention could be warranted.

According to the results of the GLMM model, two sub-groups of FSWs had higher prevalence of syphilis infection: those 35 years or older and those with less education. The higher prevalence of syphilis infection in older FSWs may be due to their increased sexual risk, including larger number of clients and higher frequency of unprotected sex [26]. Also, older FSWs are more likely to have older clients with higher prevalence of erectile dysfunction and a lower HIV risk awareness, making male condoms difficult to use and negotiate [27]. Additionally, FSWs with less education had a higher risk of syphilis infection. Research has shown that FSWs with higher levels of education are more aware of their risk for STIs and more likely to take precautions to protect themselves [28, 29]. In addition, more educated FSWs may feel more empowered to negotiate condom use with their male clients [28, 29]. They may also have better access to STI treatment compared to FSWs with less education [30, 31].

There are several limitations to our study. First, due to the high mobility and rate of loss to follow-up of low-fee FSWs, it is difficult to follow the same cohort for a long period of time. Thus, we used a cluster, controlled trial design in order to address this issue. Even though the follow-up subjects may not be the same person, the GLMM statistical analysis method used is suitable for solving such problems and account for the study design defects to some extent. Second, convenience sampling was used to recruit low-fee FSWs whom were present during the times when the venues were visited by outreach workers. There may be selection bias in the sample (e.g., newcomers to a venue were recruited but more frequent, resident FSWs were missed during the recruitment period). However, efforts were made to capture a representative sample of the low-fee FSW population, by selecting individuals from across cities at a variety of venues. More so, this study was implemented in three provinces in southwest China, where cultural and structural differences amongst low-tier FSWs may exist. Thus, these results may not be generalizable to the larger FSW community. Implementing this comprehensive intervention package in other provinces throughout China should be considered. Lastly, some behavioral variables, such as condom use and awareness of HIV related knowledge, improved in all study cities. This may be due to the enforcement of national routine HIV/STI prevention work, which may weaken the true impact of the intensive intervention measures.

## Conclusions

This study assessed the effect of a comprehensive intervention for low-fee FSWs and demonstrated that a matched-paired, community-based, comprehensive intervention was associated with declining syphilis infection. Combined intervention programs that integrate both behavioral components and biomedical components need to be developed and utilized in high-risk populations. Moreover, our study identified a more vulnerable sub-group of low-fee FSWs, indicating that they may require additional, tailored attention in future intervention plans.

## Supplementary information

**Additional file 1.** Multilingual abstracts in the five official working languages of the United Nations.

**Additional file 2.** Supplementary Material. Description of the imeplementation of the intervention activities

## Data Availability

The datasets used and/or analysed during the current study are available from the corresponding author, Zunyou Wu, Email: wuzy@263.net; wuzunyou@chinaaids.cn, on reasonable request.

## Abbreviations

AIDS: Acquired immune deficiency syndrome
CBO: Community-based organization
CDC: Center for Disease Control and Prevention
*CI*: Confidence intervals
ELISA: Enzyme-linked immunosorbent assays
FSW: Female sex worker
GLMM: Generalized linear mixed models
HIV: Human immunodeficiency virus
*OR*: Odds ratios
RMB: Renminbi (Chinese currency unit)
RPR: Rapid Plasma Reagin
STD: Sexually transmitted diseases
STI: Sexually transmitted infection
TPPA: Treponema Pallidum Particle Aggltination
USD: United States dollars

## References

[1] Chen B, Chen J, Shao Y, Hu D, Ding X, Wen Y (2019). Need for intervention Services for Promotion of condom use by female sex workers to consider size of entertainment venues: a cross-sectional study. Med Sci Monit Basic Res.

[2] Baral S, Beyrer C, Muessig K, Poteat T, Wirtz AL, Decker MR (2012). Burden of HIV among female sex workers in low-income and middle-income countries: a systematic review and meta-analysis. Lancet Infect Dis.

[3] Zhang L, Chow EP, Su S, Yiu WL, Zhang X, Iu KI (2015). A systematic review and meta-analysis of the prevalence, trends, and geographical distribution of HIV among Chinese female sex workers (2000-2011): implications for preventing sexually transmitted HIV. Int J Infect Dis.

[4] Yang H, Li X, Stanton B, Liu H, Liu H, Wang N (2005). Heterosexual transmission of HIV in China: a systematic review of behavioral studies in the past two decades. Sex Transm Dis.

[5] Qian L, Zhuang K, Henderson GE, Shenglong Q, Fang J, Yao H (2014). The organization of sex work in low and high-priced venues with a focus on the experiences of ethnic minority women working in these venues. AIDS Behav.

[6] Chu Z, Hsieh E, Rou K, Tillman J, Dong W, Feng X-X (2019). Short-term acceptability of female condom use among low-fee female sex workers in China: a follow-up study. BMC Womens Health.

[7] Chen Y, Shen Z, Morano JP, Khoshnood K, Wu Z, Lan G (2015). Bridging the epidemic: a comprehensive analysis of prevalence and correlates of HIV, hepatitis C, and syphilis, and infection among female sex workers in Guangxi Province, China. PLoS One.

[8] Zhou C, Rou K, Dong WM, Wang Y, Dong W, Zhou Y (2014). High prevalence of HIV and syphilis and associated factors among low-fee female sex workers in mainland China: a cross-sectional study. BMC Infect Dis.

[9] Chen XS, Liang GJ, Wang QQ, Yin YP, Jiang N, Zhou YJ (2012). HIV prevalence varies between female sex workers from different types of venues in southern China. Sex Transm Dis.

[10] Liu Q, Zhuang K, Henderson GE, Shenglong Q, Fang J, Yao H (2014). The organization of sex work in low- and high-priced venues with a focus on the experiences of ethnic minority women working in these venues. AIDS Behav.

[11] Cui Y, Guo W, Li D, Wang L, Shi CX, Brookmeyer R (2016). Estimating HIV incidence among key affected populations in China from serial cross-sectional surveys in 2010-2014. J Int AIDS Soc.

[12] Wariki WM, Ota E, Mori R, Koyanagi A, Hori N, Shibuya K. Behavioral interventions to reduce the transmission of HIV infection among sex workers and their clients in low- and middle-income countries. Cochrane Database Syst Rev. 2012;15(2):CD005272. 10.1002/14651858.CD005272.pub3 PMC11345029 22336811

[13] World Health Organization (WHO). Consolidated guidelines on HIV prevention, diagnosis, treatment and care for key populations. Geneva. 2016. http://www.who.int/hiv/pub/guidelines/keypopulations-2016/en/. Accessed on 1 Aug, 2019. 27559558

[14] Chuang JH, Hripcsak G, Heitjan DF (2002). Design and analysis of controlled trials in naturally clustered environments: implications for medical informatics. J Am Med Inform Assoc.

[15] Bing EG, Cheng KG, Ortiz DJ, Ovalle-Bahamon RE, Ernesto F, Weiss RE (2008). Evaluation of a prevention intervention to reduce HIV risk among Angolan soldiers. AIDS Behav.

[16] Murray DM, Varnell SP, Blitstein JL (2004). Design and analysis of group-randomized trials: a review of recent methodological developments. Am J Public Health.

[17] Kelly JA, Murphy DA, Sikkema KJ, McAuliffe TL, Roffman RA, Solomon LJ (1997). Randomised, controlled, community-level HIV-prevention intervention for sexual-risk behaviour among homosexual men in US cities. Commun HIV Prev Res Collab Lancet.

[18] Wang B, Wang QQ, Yin YP, Liang GJ, Jiang N, Gong XD (2012). The effect of a structural intervention for syphilis control among 3597 female sex workers: a demonstration study in South China. J Infect Dis.

[19] Liu J, Calzavara L, Mendelsohn JB, O'Leary A, Kang L, Pan Q (2015). Impact evaluation of a community-based intervention to reduce risky sexual behaviour among female sex workers in Shanghai, China. BMC Public Health.

[20] Black V, Williams BG, Maseko V, Radebe F, Rees HV, Lewis DA (2016). Field evaluation of standard Diagnostics' bioline HIV/syphilis duo test among female sex workers in Johannesburg, South Africa. Sex Transm Infect.

[21] Chen XS, Yin YP, Liu GG, Wei WH, Wang HC, Yu YL (2013). Outreach syphilis testing services by different health providers to female sex workers in southern China. PLoS One.

[22] Wahed T, Alam A, Sultana S, Rahman M, Alam N, Martens M (2017). Barriers to sexual and reproductive healthcare services as experienced by female sex workers and service providers in Dhaka city, Bangladesh. PLoS One.

[23] Phrasisombath K, Thomsen S, Sychareun V, Faxelid E (2012). Care seeking behaviour and barriers to accessing services for sexually transmitted infections among female sex workers in Laos: a cross-sectional study. BMC Health Serv Res.

[24] Veldhuijzen NJ, van Steijn M, Nyinawabega J, Kestelyn E, Uwineza M, Vyankandondera J (2013). Prevalence of sexually transmitted infections, genital symptoms and health-care seeking behaviour among HIV-negative female sex workers in Kigali, Rwanda. Int J STD AIDS.

[25] Liu J, Liviana C, Joshua BM, Ann O, Laiyi K, Pan QC (2015). Impact evaluation of a community-based intervention to reduce risky sexual behaviour among female sex workers in Shanghai, China. BMC Public Health.

[26] Liu H, Dumenci L, Morisky DE, Xu Y, Li X, Jiang B (2016). Syphilis among middle-aged female sex workers in China: a three-site cross-sectional study. BMJ Open.

[27] Su S, Li X, Zhang L, Lin D, Zhang C, Zhou Y (2014). Age group differences in HIV risk and mental health problems among female sex workers in Southwest China. AIDS Care.

[28] Markosyan K, Lang DL, DiClemente RJ (2014). Correlates of inconsistent refusal of unprotected sex among Armenian female sex workers. AIDS Res Treat.

[29] Meekers D, Richter K (2005). Factors associated with use of the female condom in Zimbabwe. Int Fam Plan Perspect.

[30] Mutagoma M, Nyirazinyoye L, Sebuhoro D, Riedel DJ, Ntaganira J (2017). Syphilis and HIV prevalence and associated factors to their co-infection, hepatitis B and hepatitis C viruses prevalence among female sex workers in Rwanda. BMC Infect Dis.

[31] Li Y, Detels R, Lin P, Fu X, Deng Z, Liu Y (2010). Prevalence of HIV and STIs and associated risk factors among female sex workers in Guangdong Province, China. J Acquir Immune Defic Syndr.

