# Supplementary material for: A community-based comprehensive intervention to reduce syphilis infection among low-fee female sex workers in China: a matched-pair, community-based randomized study
Source: Infect Dis Poverty. 2019 Dec 3;8:97. doi: 10.1186/s40249-019-0611-z (PMC6889532; doi:10.1186/s40249-019-0611-z)
Supplement: Supplementary file 1 — Additional file 1. Multilingual abstracts in the five official working languages of the United Nations. [file 40249_2019_611_MOESM1_ESM.pdf]

Translation of the abstract into the five official working languages of the United Nations

تدخل شامل لتقليل إصابة المومسات العاملات بأجر منخفض بمرض الزهري في الصين: دراسة مقارنة بثنائيات متطابقة وفقًا لعينة عشوائية من المجتمع

الملخص

الخلفية: إن المومسات العاملات بأجر منخفض (FSWs) معرضات بشكل كبير لفيروس نقص المناعة البشرية (HIV)/الأمراض المنقولة جنسيًا (STDs) ونشرهما في الصين. هناك حاجة ملحة لتطوير إجراءات التدخل الشاملة التي تستهدف العاملات في مجال الجنس بأجر منخفض لتقليل انتشار فيروس نقص المناعة البشرية والأمراض المنقولة جنسيًا. وأُجريت تجربة مقارنة بثنائيات متطابقة وفقًا لعينة عشوائية من المجتمع في اثني عشر مدينة في ثلاث مقاطعات في الصين.

الطريقة: تم جمع أربع مدن من نفس المقاطعة على شكل ثنائيات وحصلت المشاركات إما على حزمة تدخل (وتتضمن ترويج استعمال الواقيات الجنسية واختبارات فيروس نقص المناعة البشرية ومرض الزهري وسداد مبالغ العلاج وتقديم علاج ضد الفيروسات المرتدة أو حصلنّ على مستوى الرعاية الحالي. أُستعملت العينات الملائمة وفقًا للمكان لتعيين العاملات في مجال الجنس بأجر منخفض. أُجريت مقابلات وجهًا لوجه وأُجريت تحاليل الدم للبحث فيروس نقص المناعة البشرية ومرض الزهري ومن ثم أُجريت المتابعة على فترات دامت أربعة وعشرين شهرًا. أُستعملت النماذج الخطية المختلطة المعمَّمة (GLMM) لتقييم مدى تأثير حزمة التدخل على تقليل انتشار فيروس نقص المناعة البشرية والأمراض المنقولة جنسيًا في المومسات العاملات بأجر منخفض.

النتائج: سجلت ألف وأربع وعشرون (1024) مومسة تعمل بأجر منخفض في الاستقصاء الأساسي وسجلت ثماني مئة وأربع وعشرون (843) منهن في المتابعة. أوضحت نتائج النماذج الخطية المختلطة المعمَّمة أن انتشار مرض الزهري انخفض بنسبة تسعة وأربعين بالمائة في مجموعة التدخل مقارنة بالمجموعة التي تتلقى الرعاية الطبية المعيارية (القيمة الاحتمالية = 0.0378، نسبة الأرجحية = 0.51، 95%، مجال الثقة: 0.27-0.96). وأُكتشف أن المومسات بأجر منخفض اللواتي يبلغن خمسة وثلاثين سنة أو أكبر هن الأكثر عرضة للإصابة بمرض الزهري بنسبة مضاعفة تبلغ 2.38 مقارنة بالعاملات في مجال الجنس بأجر منخفض دون سن الخامسة والثلاثون (القيمة الاحتمالية < 0.0001، نسبة الأرجحية = 2.38، 95% مجال الثقة: 1.55-3.65). تقل مخاطر انتشار مرض الزهري بين العاملات في مجال الجنس بأجر منخفض المتعلمات بنسبة تبلغ 0.43 مقارنة بالمومسات العاملات بأجر منخفض غير المتعلمات أو ذوات مستوى تعليم أدنى (النسبة الاحتمالية < 0.05، نسبة الأرجحية = 0.43، 95% مجال الثقة: 0.63-0.93).

الاستنتاجات: توضح هذه الدراسة أن التدخلات الشاملة متصلة بانخفاض مرض الزهري بشكل ملحوظ بين المومسات العاملات بأجر منخفض. يجب النظر في ضم إجراءات التدخل السلوكية والطب الحيوي أثناء تطوير

Translated from English version into Arabic by Aalya Al-Beeshi, Revised by Shaima'a al-Fahel, through

TRANSLATORS
WITHOUT BORDERS

中国减少低档暗娼梅毒感染综合干预： 配对群组随机化研究

董薇，周楚，柔克明，吴尊友，陈军，Sarah Robbins Scott，贾曼红，周月姣，陈曦

摘要

背景：中国低档暗娼人群感染和传播艾滋病或性病的风险较高。为降低艾滋病或性病感染率，迫切需要开展针对低档暗娼人群的综合干预服务。本研究在中国三个省共选择 12 个城市开展针对低档暗娼人群的配对群组随机化干预试验。

方法：隶属同一省份的四个城市将被分别配对，每个对子里面的两个城市被随机分到干预组或对照组。干预组低档暗娼人群接受的干预服务包括安全套推广使用、艾滋病和梅毒检测促进、报销梅毒治疗费用和及时的艾滋病抗病毒治疗，对照组仅接受常规防治服务。以方便抽样的方式在暗娼活动场所招募低档暗娼人群。在基线和 24 个月后的终期随访中，分

别开展面对面问卷调查以及艾滋病病毒和梅毒血清学检测。运用广义线性混合模型来评估针对低档暗娼人群的干预措施在降低艾滋病或性病感染方面的效果。
结果：基线调查总共纳入 1024 名符合标准的研究对象，终期调查纳入 843 人。广义线性模型结果提示，干预组比常规防治组梅毒感染率降低 49% (*P* = 0.0378, *OR* = 0.51, 95% *CI*: 0.27–0.96) 。35 岁及以上的低档暗娼人群感染梅毒的风险是 35 岁以下人群的 2.38 倍 (*P* < 0.0001, *OR* = 2.38, 95% *CI*: 1.55–3.65) 。文化程度较高的低档暗娼人群感染梅毒风险是低文化程度人群的 0.43 倍 (*P* < 0.05, *OR* = 0.43, 95% CI: 0.63–0.93)。
结论：本研究结果表明综合干预服务可以有效降低低档暗娼人群的梅毒感染率。今后在开发设计针对低档暗娼人群的干预项目时需要考虑将行为学和生物学干预措施结合起来综合应用。

Translated from English version into Chinese by Wei Dong

Une intervention complète visant à réduire l'infection par la syphilis chez les travailleuses du sexe à tarifs réduits en Chine: une étude communautaire aléatoire par paire

Wei Dong, Chu Zhou, PhD, Ke-Ming Rou, Zun-You Wu, PhD**,** Jun Chen, Sarah Robbins Scott, Man-Hong Jia, Yue-Jiao Zhou and Xi Chen**Abstrait**

Contexte : Les travailleuses du sexe à tarifs réduits (FSW) courent un risque élevé de contracter et de propager le virus de l'immunodéficience humaine (VIH) et les maladies sexuellement transmissibles (MST) en Chine. Il s'avère urgent de mettre au point des mesures d'intervention complètes visant les travailleuses du sexe à tarifs réduits (FSW) afin de réduire les infections par le VIH et les MST. Un essai d'intervention aléatoire basé sur la communauté et par paire a été mené dans 12 villes de trois provinces en Chine.
Méthodes: Quatre villes de la même province ont été jumelées et les participants ont reçu soit le programme d'intervention (promotion du préservatif, dépistage du VIH et de la syphilis, remboursement des coûts de traitement de la syphilis et traitement antirétroviral gratuit, ou traitement standard actuel inclus). Un échantillonnage de commodité basée sur le lieu a été utilisé pour recruter des travailleuses du sexe à tarifs réduits (FSW). Un entretien personnel et des tests sanguins de dépistage du VIH et de la syphilis ont été réalisés à des intervalles de référence et de suivi de 24 mois. Des modèles mixtes linéaires généralisés (GLMM) ont été utilisés pour évaluer l'effet du programme d'intervention sur la réduction de l'infection à VIH et les MST chez les travailleuses du sexe à tarifs réduits (FSW).
Résultats: Un total de 1024 FSW éligibles ont été inscrites à l'enquête initiale et 843 au suivi. Les résultats des GLMM ont montré que l'infection par la syphilis était réduite de 49% dans le groupe d'intervention comparé au groupe de traitement standard actuel (*P* = 0.0378, *OR* = 0.51, 95% *CI*: 0.27–0.96). Les FSW âgées de 35 ans ou plus étaient 2.38 fois plus susceptibles de contracter la syphilis que celles âgées de moins de 35 ans (*P* < 0.0001, *OR* = 2.38, 95% *CI*: 1.55–3.65). Le risque d'infection par la syphilis chez les travailleuses du sexe à tarifs réduits (FSW) plus scolarisées était de 0.43 fois moins élevé que chez les FSW moins scolarisés (*P* < 0.05, *OR* = 0.43, 95% CI : 0.63–0.93).
Conclusions : Cette étude démontre que des interventions globales sont associées à un déclin significatif de l'infection par la syphilis chez les FSW plus scolarisées. L'intégration de mesures d'intervention à la fois comportementales et biomédicales devrait être prise en compte lors de l'élaboration de programmes pour les travailleuses du sexe à tarifs réduit (FSW)

Translated from English version into French by Natalia Sarmento, Revised by Emilie Rigault

Fourcadier, through

Комплексное вмешательство в целях снижения заболеваемости сифилисом среди низкооплачиваемых работниц секс-бизнеса в Китае: рандомизированное исследование на основе парного подхода

Вэй Дун, Чу Чжоу, Ph.D, Ке-Мин Роу, Цзун-Ты Ву, Ph.D**,**
Цзюнь Чэнь, Сара Роббинс Скотт, Ман-Хун Цзя, Юэ-Цзяо Чжоу и Си Чэнь

Аннотация

Предпосылки: низкооплачиваемые секс-работницы (СР) подвергаются высокому риску приобретения и распространения вируса иммунодефицита человека (ВИЧ)/венерических заболеваний (ЗППП) в Китае. Существует настоятельная необходимость в разработке всеобъемлющих мер вмешательства, направленных на СР, в целях сокращения числа случаев инфицирования ВИЧ/ЗППП. В 12 городах трех провинций Китая было проведено рандомизированное исследование с использованием сопоставленных пар на уровне поселений.

Методы: Четыре города из одной и той же провинции были объединены в пары и участницы получали либо пакет мер вмешательства (включая раздачу презервативов, тестирование на ВИЧ и сифилис, возмещение расходов на лечение сифилиса и бесплатную антиретровирусную терапию (АРТ), либо стандартную на этот момент медицинскую помощь. СР привлекали удобным отбором на месте. Интервью с глазу на глаз и анализ крови на ВИЧ и сифилис проводились в начале и через интервалы в 24 месяца. Для оценки эффекта от пакета мер вмешательства на снижение инфицированности ВИЧ/ЗППП в РСБ были использованы обобщенные линейные смешанные модели (GLMM).

Результаты: В базовом обследовании было охвачено в общей сложности 1024 имеющих право на участие СР и 843 СР участвовало последующих обследованиях. Результаты GLMM показали, что инфицирование сифилисом было снижено на 49% в группе вмешательства по сравнению с группой, получавшей стандартные услуги (*P* = 0,0378, *OR* = 0,51, 95% *C*I: 0,27–0,96). СР в возрасте 35 лет и старше имели в 2,38 раза больше шансов заразиться сифилисом по сравнению с лицами моложе 35 лет (*P*< 0,0001, *OR* = 2,38, 95% C*I*: 1,55–3,65). Риск заражения сифилисом среди более образованных СР был в 0,43 раза меньше, чем у лиц с низким уровнем образования (*P*< 0,05, *OR* = 0,43, 95% CI: 0,63–0,93).

Выводы: данное исследование демонстрирует, что комплексные вмешательства связаны со значительным снижением заболеваемости сифилисом среди низкоуровневых СР. При разработке программ для низкооплачиваемых СР должна быть рассмотрена интеграция как поведенческих, так и биомедицинских мер вмешательства.

Translated from English version into Russian by Michael Orlov, Revised by Alexander Somin, through

Una intervención exhaustiva, para reducir infección por sífilis, entre mujeres prostitutas con baja remuneración, en China: un estudio aleatorio de las parejas, basado en la comunidad

Wei Dong, Chu Zhou, Ke-Ming Rou, Zun-You Wu, PhD**,** Jun Chen, Sarah Robbins Scott, Man-Hong Jia, Yue-Jiao Zhou y Xi Chen

Resumen

Antecedentes: las trabajadoras prostitutas (FSWs; por sus siglas en inglés), con una baja remuneración, corren un alto riesgo de contraer y propagar el virus de la inmunodeficiencia humana (VIH) y enfermedades de transmisión sexual, en China. Existe una necesidad urgente de elaborar medidas de intervención exhaustivas, destinadas a las FSWs, con baja remuneración, para reducir las infecciones de VIH / ETS. En 12 ciudades, situadas en tres provincias de China, se llevó a cabo un ensayo de intervención aleatoria basado en la comunidad.

Método: Se emparejaron cuatro ciudades de la misma provincia y los participantes recibieron el paquete de intervención (incluida la promoción del condón, las pruebas de VIH y de sífilis, el reembolso de los costos del tratamiento de la sífilis y una terapia antirretroviral gratuita (ART) o el estándar de atención actual). Se utilizó un muestreo de conveniencia, para reclutar a las FSWs. Se realizó una entrevista cara a cara y un análisis de sangre de sífilis y VIH, al inicio y a intervalos del seguimiento de 24 meses de duración. Se utilizaron modelos lineales mixtos generalizados (GLMM), para evaluar el efecto del paquete de intervención, a fin de reducir la infección por VIH / ETS, en las FSW..

Resultados: Se inscribieron un total de 1024 FSW elegibles, en la encuesta de referencia y 843 en el seguimiento. Los resultados de GLMM mostraron que la infección por sífilis se redujo en un 49%, en el grupo de intervención, en comparación con el grupo de atención estándar actual (P = 0.0378, OR = 0.51, IC 95%: 0.27–0.96). Los FSW, de 35 años o más, tenían 2,38 veces más probabilidades de contraer la sífilis, en comparación con los menores de 35 años (P <0,0001, OR = 2,38, IC 95%: 1,55–3,65). El riesgo de infección por sífilis, entre los FSW más educados, fue 0.43 veces menor que aquellos con niveles más bajos de educación (P <0.05, OR = 0.43, IC 95%: 0.63–0.93).

Conclusiones: Este estudio demuestra que las intervenciones integrales están asociadas con una disminución significativa de la infección por sífilis, entre las FSW, con bajo nivel. Se debe considerar la integración de medidas de intervención conductual y biomédica, al desarrollar programas para FSW de bajo costo.

Translated from English version into Spanish by Ana Gonzalez, Revised by Maria Luz Puerta, through

TRANSLATORS
WITHOUT BORDERS
