# Supplementary material for: A community-based comprehensive intervention to reduce syphilis infection among low-fee female sex workers in China: a matched-pair, community-based randomized study
Source: Infect Dis Poverty. 2019 Dec 3;8:97. doi: 10.1186/s40249-019-0611-z (PMC6889532; doi:10.1186/s40249-019-0611-z)
Supplement: Supplementary file 2 — Additional file 2. Supplementary Material. Description of the imeplementation of the intervention activities [file 40249_2019_611_MOESM2_ESM.pdf]

Supplementary Materials

Section 1. Implementation of Intervention Activities

Over a period of 27 months, the quantitative indicators of measures in the cities receiving the intervention were:

1) Condom promotion: a popular brand of condoms was identified according to the information obtained from pre-research. Trained local outreach workers offered this brand of condom to the FSWs once a month and provided training on proper use, safe sex behaviors, and condom use negotiation. By the end of the study, we purchased and sent 20,6560 brand condoms to each city implementing the intervention package.

2) Intensive HIV and syphilis testing: local outreach workers encouraged low-fee FSWs tested more than three months prior to get tested for HIV and syphilis. During the overall research period, the number of people tested in the intervention cities was about 2,500, which was approximately 20% more than in the current care cities (2,000 people tested).

3) Reimbursement for syphilis infection treatment: one local STI treatment clinic was selected for syphilis-positive participants' referral and treatment. Each patient was treated following the national standard program for syphilis treatment and reimbursed for the cost of her syphilis treatment. A total of 180 syphilis infected patients were found in the intervention cities, of those 132 (73.3%) were successfully referred to the STI clinic for further physical examination and treatment. The other 48 patients were absent from treatment because of either loss to follow-up or not willing to be treated.

4) Timely ART for HIV/AIDS: the study participants with confirmed HIV positive results were offered immediate ART initiation irrespective of CD4+ T cell count. For their first visit, the local outreach staff accompanied the FSWs to the HIV/AIDS clinic and helped them complete their physical examination and enrollment in the HIV/AIDS treatment scheme. A total of ninety HIV positive cases were found during the study period. Among them, 33 were in intervention group, and 57 were in current standard of care group. By the end of 2015, 60.6% cases (20/33) in intervention group were receiving ART successfully, which was significantly higher than the current care group (24.6%, 14/57).
